# Consensus Paper: The Role of the Cerebellum in Perceptual Processes

**DOI:** 10.1007/s12311-014-0627-7

**Published:** 2014-12-06

**Authors:** Oliver Baumann, Ronald J. Borra, James M. Bower, Kathleen E. Cullen, Christophe Habas, Richard B. Ivry, Maria Leggio, Jason B. Mattingley, Marco Molinari, Eric A. Moulton, Michael G. Paulin, Marina A. Pavlova, Jeremy D. Schmahmann, Arseny A. Sokolov

**Affiliations:** 1 Queensland Brain Institute, The University of Queensland, St. Lucia, Queensland Australia; 2 Department of Radiology and Athinoula A. Martinos Center for Biomedical Imaging, Massachusetts General Hospital and Harvard Medical School, Charlestown, MA USA; 3 Department of Diagnostic Radiology, Medical Imaging Centre of Southwest Finland, Turku University Hospital, Turku, Finland; 4 Numedon Inc., Pasadena, CA USA; 5 Department of Physiology, McGill University Montreal, Montreal, Canada; 6 Service de NeuroImagerie, CHNO des Quinze-Vingts, UPMC Paris 6, Paris, France; 7 Department of Psychology, University of California, Berkeley, CA USA; 8 Department of Psychology, Sapienza University of Rome, Rome, Italy; 9 I.R.C.C.S. Santa Lucia Foundation, Rome, Italy; 10 Pain/Analgesia Imaging Neuroscience (P.A.I.N.) Group, Department of Anesthesia, Boston Children’s Hospital, Center for Pain and the Brain, Harvard Medical School, Waltham, MA USA; 11 Department of Zoology, University of Otago, Otago, New Zealand; 12 Department of Biomedical Magnetic Resonance, Medical School, Eberhard Karls University of Tübingen, Tübingen, Germany; 13 Ataxia Unit, Cognitive Behavioral Neurology Unit, Laboratory for Neuroanatomy and Cerebellar Neurobiology Department of Neurology, Massachusetts General Hospital and Harvard Medical School, Boston, MA USA; 14 Département des Neurosciences Cliniques, Centre Hospitalier Universitaire Vaudois (CHUV), Lausanne, Switzerland

**Keywords:** Audition, Biological motion, Cerebellum, Connectivity, Evolution, fMRI, Pain, Perception, Prediction, Single-unit recording, Self-motion, Sequencing, State estimation, Timing, Vision

## Abstract

Various lines of evidence accumulated over the past 30 years indicate that the cerebellum, long recognized as essential for motor control, also has considerable influence on perceptual processes. In this paper, we bring together experts from psychology and neuroscience, with the aim of providing a succinct but comprehensive overview of key findings related to the involvement of the cerebellum in sensory perception. The contributions cover such topics as anatomical and functional connectivity, evolutionary and comparative perspectives, visual and auditory processing, biological motion perception, nociception, self-motion, timing, predictive processing, and perceptual sequencing. While no single explanation has yet emerged concerning the role of the cerebellum in perceptual processes, this consensus paper summarizes the impressive empirical evidence on this problem and highlights diversities as well as commonalities between existing hypotheses. In addition to work with healthy individuals and patients with cerebellar disorders, it is also apparent that several neurological conditions in which perceptual disturbances occur, including autism and schizophrenia, are associated with cerebellar pathology. A better understanding of the involvement of the cerebellum in perceptual processes will thus likely be important for identifying and treating perceptual deficits that may at present go unnoticed and untreated. This paper provides a useful framework for further debate and empirical investigations into the influence of the cerebellum on sensory perception.

## Introduction

For 150 years, functional analyses of the cerebellum have focused on the role of this subcortical structure in the control and coordination of movement. In the past 30 years, however, clinical, experimental, and neuroimaging studies have provided compelling evidence for the involvement of the cerebellum in task domains as diverse as memory, language, and emotion. Crucially, several lines of evidence suggest that the cerebellum has an influence on perceptual functions. Observations from anatomical and electrophysiological studies in monkeys and cats indicate the existence of cerebellar connections with visual- and auditory-related cortices. Moreover, clinical reports in humans have revealed that both focal and diffuse lesions of the cerebellum lead to a wide range of sensory impairments. While damage to the cerebellum does not cause a complete loss of sensory function, it is apparent that several sensory and perceptual processes are affected, such as motion and time perception, or the ability to recognize perceptual sequences.

In this consensus paper, we summarize key findings and concepts with the aim of demonstrating and explaining the cerebellar influence on perceptual tasks. To this end, we have gathered contributions from 14 experts in complementary fields of psychology and neuroscience, providing a range of different and sometimes controversial viewpoints. We believe that a new consensus that draws on and integrates the ideas presented here will help unravel the enigmatic role or influence of the cerebellum in perceptual processing. The review begins with a succinct overview of the anatomical connections of the cerebellum with sensory and perceptual areas in the cerebrum by Dr. Schmahmann. Dr. Habas then provides an evaluation of the functional connections between the cerebellum and cerebral perceptual systems, drawing on studies using modern neuroimaging techniques. Dr. Paulin provides an evolutionary and comparative perspective on cerebellar involvement in perceptual functions. Evidence for a cerebellar role in visual and auditory processing is summarized by Drs. Baumann and Mattingley, followed by a commentary from Drs. Pavlova and Sokolov on visual processing of biological motion. Dr. Cullen writes on the critical function of the cerebellum in self-motion perception. Evidence for a role of the cerebellum in pain perception is reviewed by Drs. Borra and Moulton. Dr. Ivry presents a hypothesis and data to suggest that the cerebellum acts as a timing device for motor and non-motor processes. Drs. Leggio and Molinari present evidence for a model that posits a central role for the cerebellum in the detection and prediction of perceptual sequences. The review closes with a contribution from Dr. Bower, who suggests that the cerebellum is not itself involved in perceptual processing, but instead, its influence on perception as well as motor control, is indirect through its role in monitoring and adjusting the acquisition of sensory data.

## Anatomical Circuits Relevant to the Role of the Cerebellum in Perception (J.D. Schmahmann)

The cerebellar role in perception is predicated on the fact that it is an essential node in the distributed neural circuits subserving sensorimotor, autonomic, and cognitive function as well as emotional processing. The following is a summary of these pathways and connections. For earlier comprehensive reviews and original citations, please see Schmahmann [1–3] and Schmahmann and Pandya [4].

### Peripheral Afferents

Auditory and visual inputs are conveyed from primary sensory receptors to vermal lobules VI and VII [5], and visual inputs also reach the dorsal paraflocculus. Spinocerebellar tracts terminate in the sensorimotor cerebellum in the anterior lobe and lobule VIII [6], while vestibular afferents target lobule X [7]. Climbing fibers from the sensorimotor-recipient inferior olivary nuclei project to the sensorimotor cerebellum; the principal olivary nucleus is devoid of peripheral inputs and is linked with the cognitive cerebellum in the posterior lobe (see [3]).

### Cerebrocerebellar Pathways

Cerebellar connections with the cerebral cortex include two-stage feedforward and feedback loops with obligatory synapses in the pons and thalamus. The top-down circuit is corticopontine–pontocerebellar and the bottom-up is cerebellothalamic–thalamocortical.

### Corticopontine Projections

Knowledge of the corticopontine projections provides critical insights into the nature of the information to which the cerebellum has access. Projections arise from neurons in layer Vb of sensorimotor regions in the precentral, premotor, and supplementary motor area, primary somatosensory cortices, and the rostral parietal lobe [8–11]. Studies in stroke patients also show topography of motor function in the human pons [12].

**Fig. 1:**
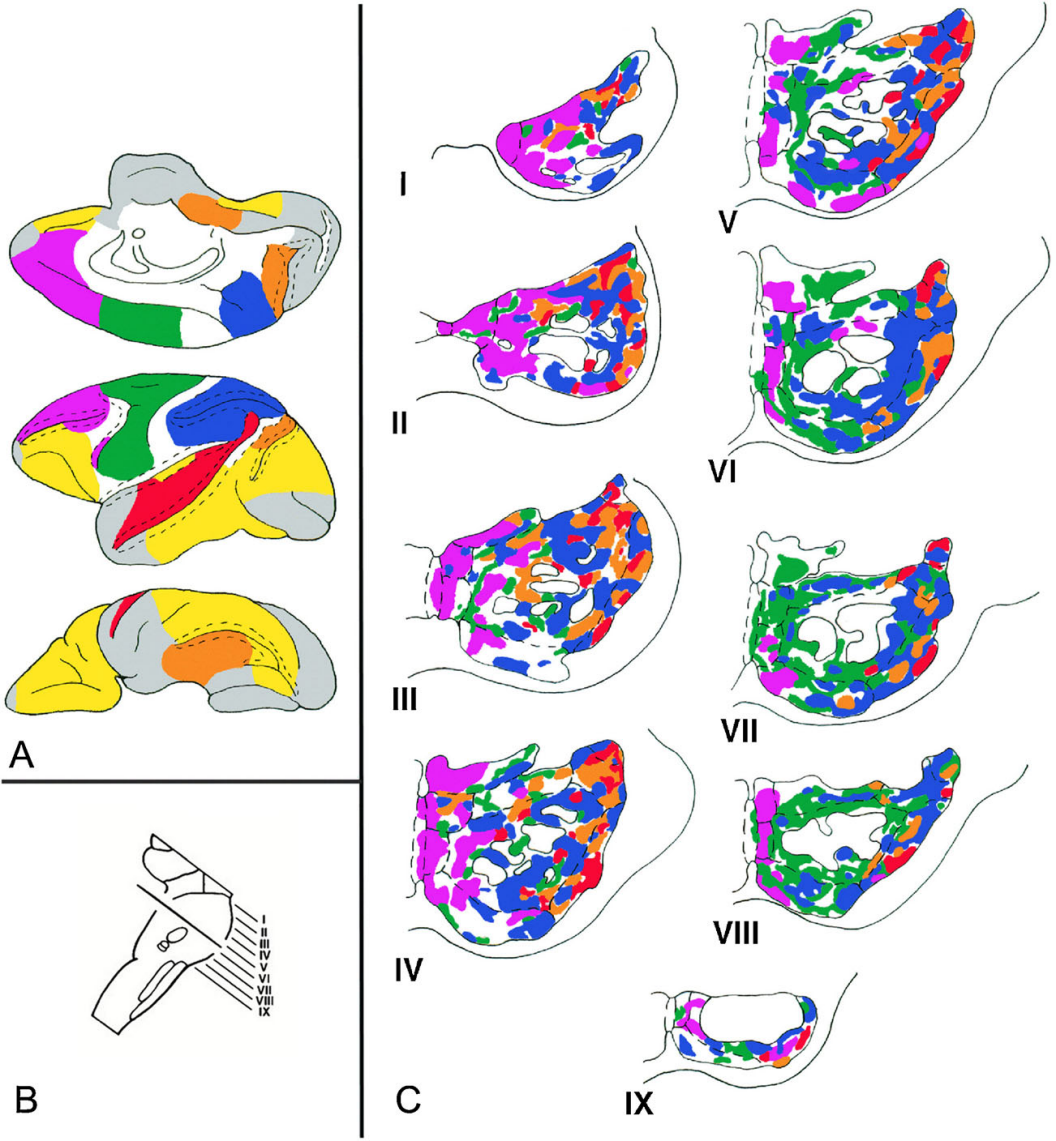
Composite color-coded summary diagram illustrating the distribution within the basis pontis of rhesus monkey of projections derived from association and paralimbic cortices in the prefrontal (*purple*), posterior parietal (*blue*), superior temporal (*red*), parastriate, and parahippocampal regions (*orange*), and from motor, premotor and supplementary motor areas (*green*). **a** Medial, lateral, and orbital views of the cerebral hemisphere from which the projections are derived. **b** Plane of section through the pons from which the rostrocaudal levels of the pons I through IX are taken. **c** Patterns of termination within the nuclei of the basis pontis. Other cerebral areas known to project to the pons are depicted in *white*. Cortical areas with no pontine projections are shown in *yellow* (from anterograde and retrograde studies) or *gray* (from retrograde studies). *Dashed lines* in the hemisphere diagrams represent sulcal cortices. *Dashed lines* in the pons diagrams represent pontine nuclei; *solid lines* depict corticofugal fibers (from [1] and [13])

Considerable corticopontine projections are derived also from the prefrontal cortex, multimodal regions of the posterior parietal and temporal lobes, paralimbic cortices in the cingulate and posterior parahippocampal gyrus, and visual association cortices in the parastriate region, supporting multimodal, supramodal, and limbic related functions necessary for perception (Fig. 1).

Prefrontopontine projections arise from dorsolateral and dorsomedial convexities concerned with attention and conjugate eye movements (area 8), spatial attributes of memory and working memory (area 9/46d), planning, foresight, and judgment (area 10), motivational behavior and decision-making capabilities (areas 9 and 32), and from areas 44 and 45 homologous to language areas in human [13].

Posterior parietal association cortices are critical for directed attention, visual–spatial analysis, and vigilance in the contralateral hemispace; lesions are associated with complex behavioral manifestations. The superior parietal lobule concerned with multiple joint position sense, touch, and proprioceptive impulses projects throughout central and lateral regions of the rostrocaudal pons. The caudal inferior parietal lobule implicated in the neglect syndrome favors the rostral half of the pons in the lateral and dorsolateral regions [10].

Auditory association areas in the superior temporal gyrus and supratemporal plane are connected with the lateral and dorsolateral pontine nuclei. Cortices in the upper bank of the superior temporal sulcus activated during face recognition tasks project to the lateral, dorsolateral, and extreme dorsolateral pontine nuclei [14]. Motion-sensitive temporal lobe areas MT (middle temporal), FST (fundus of the superior temporal sulcus), and MST (medial superior temporal) also have pontine connections [15], but inferotemporal cortex including the rostral lower bank of the superior temporal sulcus, which is relevant for feature discrimination, has no pontine efferents. Thus, the dorsal visual (where) stream concerned with motion analysis and visual–spatial attributes of motion participates in the cerebrocerebellar interaction, but the ventral visual (what) stream governing visual object identification does not. Parastriate projections from occipitotemporal and occipitoparietal regions also respect the dorsal–ventral dichotomy. The medial and dorsal prelunate regions project to the pons (dorsolateral, lateral, and lateral aspect of the peripeduncular nuclei most heavily), but ventral prelunate cortices and inferotemporal regions do not [16]. Projections from the temporal lobe homologue of the Wernicke language area in human, together with those from the monkey homologue of Broca’s area, are relevant in the light of cerebellar activation during functional neuroimaging studies of language [17, 18] and in disorders of language following cerebellar lesions [19, 20].

Paralimbic projections arise from posterior parahippocampal gyrus important for spatial attributes of memory, directed to lateral, dorsolateral, and lateral peripeduncular nuclei. Cingulate cortex projections arise from motor areas in the depth of the cingulate sulcus [21] and from areas concerned with motivation and drive in rostral and caudal cingulate areas [22]. The anterior insular cortex, important for autonomic systems and pain modulation also has pontine connections [9]. Projections arise also from multimodal deep layers of the superior colliculus and medial mammillary bodies involved in memory and emotion [23]. The hypothalamus, critical for autonomic control and limbic behaviors, has direct reciprocal connections with the cerebellum [24].

Corticopontine projections are arranged with topographic specificity. Sensorimotor terminations are more caudally situated; association areas project more rostrally. Terminations occur in multiple patches forming interdigitating mosaics. The significance of associative corticopontine inputs in human compared with monkey is underscored by enlargement in human of the medial part of the cerebral peduncle conveying prefontopontine fibers [25], reflecting evolutionary pressure in which interconnected systems evolve in concert with each other.

### Pontocerebellar Projections

The caudal pons sends sensorimotor-related information to the cerebellar anterior lobe. Rostral pontine nuclei convey cognitively relevant information to the posterior cerebellum: medial pontine projections from prefrontal cortices to crus I and to crus II, and medial, ventral, and lateral pons conveying information from parietal association cortices to crus I, crus II, and lobule VIIB. These anatomical studies extend earlier physiological conclusions that parietal and prefrontal cortices are functionally related mainly to crus I, crus II, and the paramedian lobule of the cerebellum [26]. In the pontocerebellar projection, each cerebellar folium receives input from a unique complement of pontine cell groups, some of which are widely separated [1, 27]. The pattern of diverging corticopontine projections and converging pontocerebellar projections led to the suggestion that information from one cerebral cortical area is distributed to numerous sites in the cerebellar cortex [27], although trans-synaptic viral tract tracing studies reveal that anterograde projections through the medial pons are directed to focal areas in crus I and crus II [28].

### Cerebellar Feedback

**Fig. 2:**
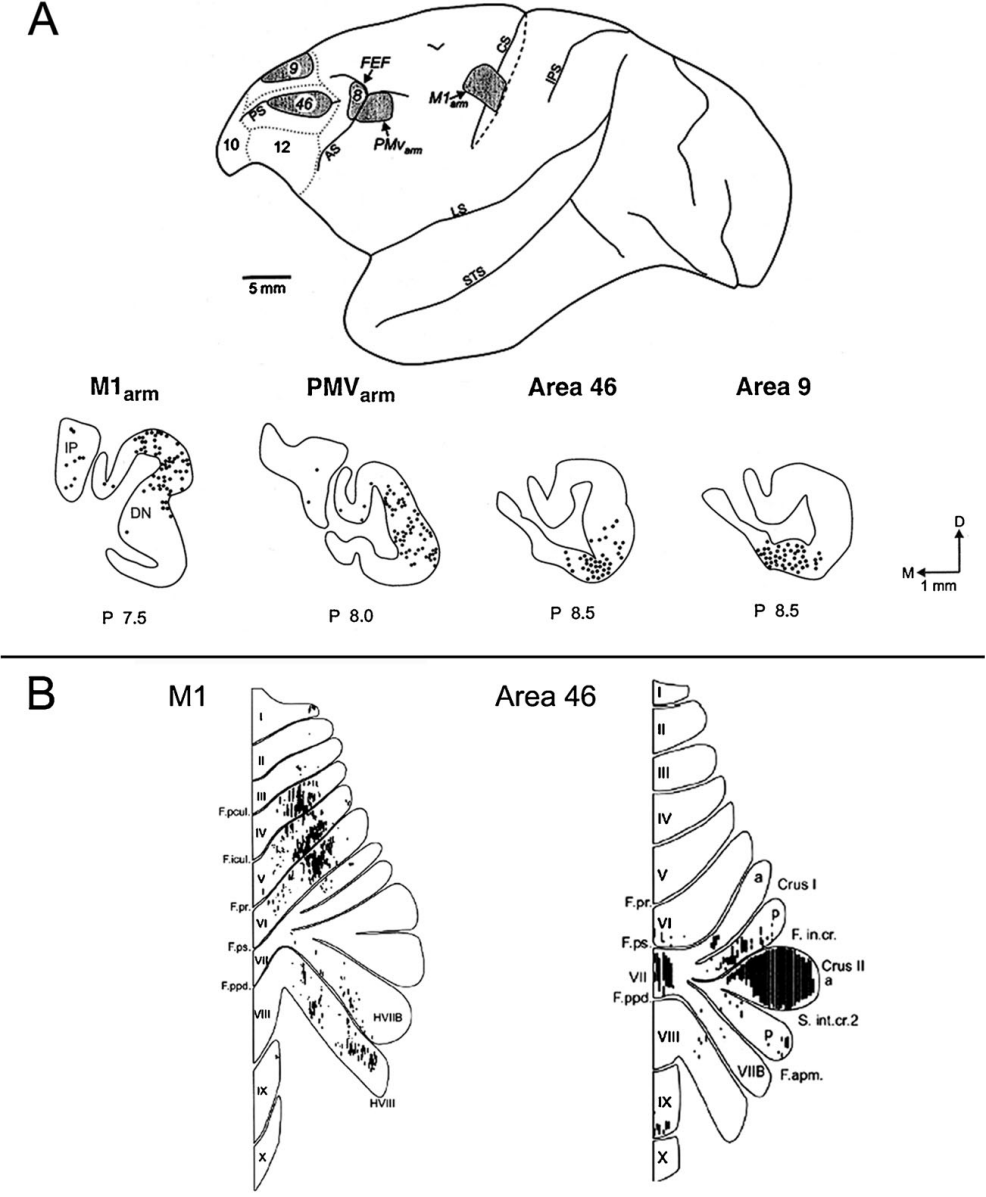
**a** Diagram of the lateral view of a cebus monkey brain (*top*) to show the location of injections of McIntyre-B strain of herpes simplex virus I in the primary motor cortex arm representation (M1arm), ventral premotor cortex arm representation (PMVarm), and in the prefrontal cortex in areas 9 and 46. The resulting retrogradely labeled neurons (*below*) in the cerebellar interpositus nucleus (IP) and dentate nucleus (DN) are indicated by *solid dots* and show the dorsal–ventral dichotomy in dentate projections to motor versus prefrontal cortices. Adapted from [29]. **b** Representation on flattened views of the cerebellum of the input–output organization of cerebellar loops with motor cortex M1 (*left*) and area 46 (*right*) revealed using anterograde and retrograde strains of rabies virus as tract tracer. M1 is interconnected with lobules IV to VI; prefrontal cortical area 46 is linked predominantly with crus II. Adapted from [28]

Purkinje cells convey the output of the cerebellar cortex to the deep cerebellar nuclei (DCN), which send projections back to the brainstem, or to the cerebral cortex via the thalamus. The cerebellar cortex–DCN–thalamus–cerebral cortex feedback loop is arranged so that motor related interpositus nuclei (globose and emboliform in human) send efferents from cerebellar anterior lobe motor areas to the cerebral sensorimotor regions, whereas the ventral dentate sends information from the cerebellar posterior lobe to cerebral association areas—prefrontal, posterior parietal, and others [28, 29] (see Fig. 2). The cerebellar vermis and fastigial nucleus are linked with brainstem and thalamic structures concerned not only with vestibular and oculomotor control, posture, and equilibrium, but also with autonomic and paralimbic cerebral areas, consistent with the notion of the vermis and fastigial nucleus as the limbic cerebellum [3].

### Synthesis

Against the backdrop of the heterogenous and topographically arranged connections of the cerebellum with the rest of the neuraxis stands the essentially constant architecture of the cerebellar cortex. This dichotomy is the basis of the dysmetria of thought theory, which poses that a constant computation—the universal cerebellar transform—is applied to multiple domains of neurological function subserved by the distributed neural circuits of which cerebellum is an integral node [3]. The anatomical connections that link the cerebellum with both the external and the internal worlds thus provide the critical neural substrates of the putative cerebellar role in perception. These conclusions from tract tracing studies in the monkey are supported by resting state functional connectivity magnetic resonance imaging (MRI; [30]) and task-based functional MRI studies in humans [18], as well as by clinical investigations in patients with cerebellar damage [19].

## Resting-State Functional Connectivity Between Cerebellum and Sensory Systems (C. Habas)

Measurement of human brain resting-state activity with MRI has allowed us to precisely determine the functional connectivity (FC) between specific zones of the cerebellum and the rest of the brain. FC is based on temporal correlations between spontaneous, low-frequency (0.01–0.1 Hz) fluctuations of the blood-oxygen-level-dependent (BOLD) signal at rest between functionally and anatomically linked cerebral areas [31]. Two main statistical methods are used to compute resting-state functional maps passing through the cerebellum [32]: (1) independent component analysis, which is used to identify multiple temporally cohesive, spatially distributed networks and (2) regression analysis of activity in a region of interest against that of the remainder of the brain. These methods have contributed to distinguish two anatomo-functional parts of the cerebellum [33–35]: a sensorimotor region (lobules V–VI and VIII) and a prominent multimodal cognitive and limbic region (lobule VIIA, especially crus I and II, with adjoining parts of lobule VI and VIIB, and lobule IX). The sensorimotor cerebellum corresponds predominantly to sensory parts of its multiple somatotopic maps that receive exteroceptive and proprioceptive inputs from spinal, trigeminal, and somatosensory cortical afferents, and send outputs to motor areas in order to control, guide, and correct ongoing movements. At least three somatotopic representations have been reliably described: the first in lobules IV–VI, the second in lobules VIIb–VIII, and a third in lobules VI–VIIA [36].

Discrepant results, however, were obtained for the visual and auditory cerebellum. O’Reilly and colleagues [34] found functional coherence between visual area MT and superior temporal gyrus, including auditory primary and associative zones, with cerebellar lobules V-VI-VIII and lobules V–VI, respectively. Buckner and colleagues, however, failed to detect any functional connectivity between auditory cortex and cerebellum [30, 33]. The proximity between the occipital lobe and the underlying cerebellar cortex has been proposed as a possible explanation of the discrepancy between these data. However, Sokolov et al. [37] (see also the section by Drs. Sokolov and Pavlova, “Cerebellar Involvement in Biological Motion Processing (A.A. Sokolov and M.A. Pavlova)”) found, using DTI, structural interconnection between cerebellar crus I and right superior temporal sulcus (STS), in agreement with a previous seed-based functional connectivity result which showed functional coherence between STS and cerebellar lobules VI/VIIA [38]. It is noteworthy that no functional link was found in these two studies between cerebellum and primary visual cortex (BA 17) in line with previous animal studies. Notwithstanding, using cerebellar seed-based functional connectivity, Sang and colleagues [39] found correlations between visual networks and hemispheric lobules I–VI and vermal lobules VIIb–IX, as well as auditory networks and hemispheric lobules VI-VIIb-VIIIa. Ding et al. [40] also identified decreased functional connectivity between visual cortex (BA 17) and cerebellum (crus I and II, vermis of lobules VI–VII and tonsilae) when they compared ambliopic patients with healthy subjects. One possibility would be that amblyoply first induced diminished connectivity between primary visual cortex and interconnected parietal (BA 40) and prefrontal (BA 6/8) cortices, and that this altered connectivity indirectly affected the cerebellum via the prefronto-parieto-pontine pathway.

The cerebellum is also involved in the limbic ‘salience network,’ mainly encompassing insula, frontal operculum, medial prefrontal cortex, and hypothalamus [35], and in charge of interoceptive and autonomic processing [41]. Therefore, it could be hypothesized that cerebellar zones belonging to the salience network (lobules VI, VIIA, and VIIB) process interoceptive data. Paravermal and vermal lobule VI may constitute a specific node receiving exteroceptive and interoceptive data, since it has been found active during emotional responses such as disgust [42]. In conclusion, functional connectivity mainly confirms previous results acquired with histological tracking and electrical stimulation and adds some new insights: the ‘sensory’ cerebellum is mainly part of the sensorimotor (and vestibular) cerebellum and may also comprise areas that process visual, auditory, and interoceptive signals. Finally, there may be two distinct roles for the cerebellum in perceptual tasks. The first involves the ‘sensory’ cerebellum for perceptual analysis, cancellation, and anticipation based on internal models during, for instance, fine exploratory movements. The second involves the polymodal ‘executive’ cerebellum, which is associated with working memory, attention, and decision-making processes for conscious elaboration of the mental representation of a perceived object [43].

## Evolutionary Perspectives on Cerebellar Function (M.G. Paulin)

Early in the twentieth century, studies of brain-damaged soldiers led to a consensus that cerebellum is dedicated to motor control, because focal cerebellar ablation led to obvious motor deficits without obvious perceptual deficits [44]. Late in the twentieth century, human functional imaging studies revealed that the cerebellum is actively engaged in a variety of cognitive, perceptual, and behavioral tasks, even when subjects are not moving [45].

In the middle of the twentieth century, the gigantocerebellum of weakly electric fish stood out as an anomaly because, in these animals, the cerebellum is evidently involved in tracking objects using the electric sense [46–48]. But comparative anatomical, physiological, and behavioral evidence indicated that this is not an anomaly. Across all vertebrates, the cerebellum seems to have a primary role in motion analysis and motion prediction, with a role in motor control a consequence of this perceptual capability, analogous to the role of dynamical state estimators in artificial control systems [49].

The theory that cerebellum is a neural analogue of a dynamical state estimator simplifies and generalizes the theory that cerebellum is engaged in motor control. An animal needs to determine the kinematic state of its own body in order to control movements, and to perceive and dynamically interact with other objects and organisms. In particular, active sensing and exploratory behavior is critically dependent on accurate information about the configuration and motion of sense organs during sensory acquisition [47]. It has been shown in human and animal studies that the cerebellum plays a crucial role in active sensory acquisition [50, 51]. Other tasks that have been shown to involve the cerebellum in humans also seem to require dynamical state estimation [52–59].

The cerebellum is a characteristic of vertebrates, but cephalopod molluscs (squid and octopus) appear to have evolved a cerebellum independently. The cephalopod cerebellum receives visual and vestibular sense data and is involved in whole-body and oculomotor stabilization during locomotion [60–62]. Cephalopods are the only agile predators among molluscs.

Cerebellar-like structures occur in a number of animal phyla. These are distinguished from the ‘cerebellum proper’ by a lack of climbing fibers and a lack of direct projections to motor and premotor structures. The most well-known cerebellar-like structures are electrosensory and lateral-line mechanosensory nuclei in fishes [63, 64], but they are found in many vertebrates including humans [65]. They are involved in removing distortions from external signal sources caused by an animal’s own activity. Thus, in electroreception, the cerebellum is involved in sensing external targets by exploiting distortions in signals generated by the animal’s own activity, while cerebellar-like circuits are involved in sensing external targets by eliminating distortions of target signals caused by the animal’s own activity.

Cerebellar-like circuits have been reported among arthropods, onychophorans, and polychaete annelids. These invertebrates are all active foragers, with appendages that support arrays of sensilla [66]. Cerebellar-like structures in insects may be involved in orientation and navigation [67]. They seem to be more prominent in species like honeybees, which use their antennae as active probes, than in moths whose antennae are passive receivers [66].

The cerebellar cortical circuit common to the cerebellum and cerebellar-like circuits has apparently evolved independently in at least five groups of animals: vertebrates, cephalopod molluscs, arthropods, onychophorans, and polychaete annelids. All species in which cerebellar and/or cerebellar-like circuits have been reported are motile and sufficiently large that their kinematics is influenced by inertia, and they interact with other such animals. Inertia constrains how the kinematic state (position, configuration, and rates of change) of an object changes as a function of applied force, such that, if an object has inertia, then information about its kinematic state can be used to predict its future position and configuration at least in the short term. This is not true of animals (or indeed objects of any kind) whose mass is small or drag is large relative to applied forces [68]. Animals that have evolved cerebellar(-like) circuits are, therefore, animals for which probabilistic inference about the kinematic states of self and others is both possible and useful. The fact that this group includes disparate, unrelated species indicates that the genetic and developmental capacity for cerebellar(-like) circuits may be shared by all animals with nervous systems and that it has been co-opted by evolution whenever there has been an ecological opportunity for animals capable of dynamic motion prediction and control [69]. More generally, the ability to predict state trajectories of dynamical systems from observations provides a core capability that may underpin a wide variety of perceptual, cognitive, and motor tasks [70].

Until a few years ago, the Kalman filter was the only known practical algorithm for dynamical state estimation [71]. It assumes linear target dynamics, an assumption that does not hold for mechanical linkages like human and animal bodies. Newer algorithms based on drawing random samples from probability distributions defined by observations are able to track states of high-dimensional nonlinear systems [72]. These algorithms can be implemented using spiking neurons, in which a spike at a particular location in a network represents a sample at a particular location in the state space of the system tracked by the network [73, 74]. There is growing evidence that neurons use Bayesian Monte-Carlo algorithms of this kind to implement decisions and actions [75–83].

## The Role of the Cerebellum in Visual and Auditory Processing (O. Baumann and J.B. Mattingley)

Over the last decade, hypotheses of human cerebellar function have undergone dramatic revisions [84]. Of these, perhaps the most intriguing is the proposal that the cerebellum plays a role in sensory processes. In the following, we review evidence for cerebellar involvement in visual and auditory perception.

Cerebellar responses to auditory and visual stimulation were described in the 1940s. Snider and Stowell [85] recorded electrical responses in the cerebellar cortex of 150 anesthetized cats, evoked by acoustic clicks as well low-intensity light flashes. Using this approach, they revealed the existence of distinct, but partially overlapping cerebellar regions, predominantly in vermal lobule VII and hemispheric lobules VI, that were differentially activated for visual stimuli and auditory stimuli. In the 1980s, several laboratories started to use neuronal tracers to examine cerebrocerebellar projections in non-human primates and discovered that visual as well as auditory association areas are anatomically connected with the cerebellum [2] (see also the section by Dr. Schmahmann, “Anatomical Circuits Relevant to the Role of the Cerebellum in Perception (J.D. Schmahmann)”). Interestingly, while cerebellar connections were found for dorsal visual stream areas, which are known to underlie motion analysis, this was not the case for ventral visual stream areas, which are involved in visual object recognition. This finding suggests that the cerebellum is particularly involved in processing dynamic (i.e., time varying) visual information.

The first evidence in humans for a cerebellar involvement in visual processes derives from work undertaken by Ivry and Diener, who found that cerebellar patients were impaired in making judgments of the velocity of moving stimuli, whereas elementary visual functions remained intact [86]. These findings were later corroborated and extended by Thier and Haarmeier, who reported that patients with cerebellar lesions were also impaired in detecting and discriminating moving visual signals in the presence of visual noise [87]. Similarly, it was found that cerebellar lesions can disturb auditory processing, by significantly increasing thresholds in duration [88] and pitch discrimination tasks [57].

**Fig. 3:**
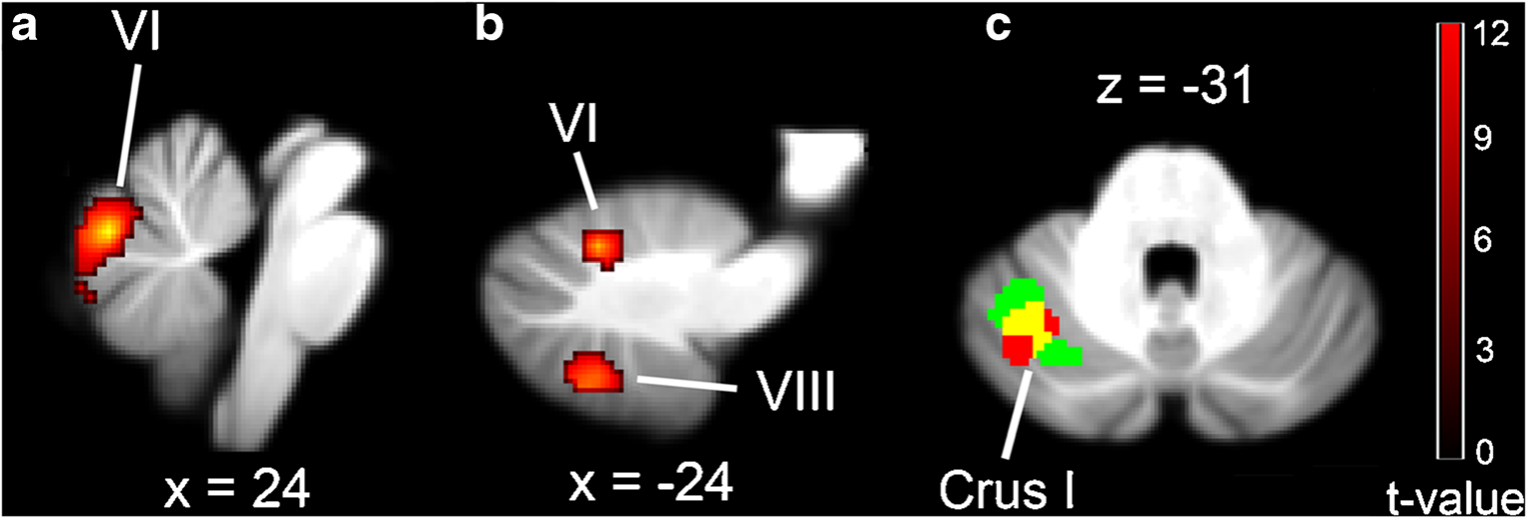
MR brain slices showing distinct set of cerebellar regions that were differentially activated for: **a** visual stimuli and **b** auditory stimuli, as well as **c** showing a negative linear relationship between fMRI signal and motion signal strength (*red shading* represents activity for the visual motion condition; *green shading* represents activity for the auditory motion condition; *yellow shading* indicates activation overlap between the visual and auditory conditions). Figure reproduced with permission from [89]

Despite evidence of a sensory processing role for the cerebellum, the exact manner in which visual and auditory information is represented in the human cerebellum remains unclear. To address this issue, we used functional magnetic resonance imaging (fMRI) to monitor neural activity within the cerebellum while participants were engaged in a task that required them to determine the direction of a visual or auditory motion signal in noise [89]. In the visual motion task, vermal lobule VI and right-hemispheric lobule X were active (see Fig. 3a), whereas in the auditory motion task, activity was elevated in hemispheric lobules VI and VIII (see Fig. 3b). Interestingly, for both auditory and visual motion tasks, activity within left crus I increased as the strength of the motion signal decreased (see Fig. 3c), suggesting that the recruitment of the cerebellum is related to the perceptual demands of a task. These findings are consistent with results from a positron emission tomography study in which similar regions of cerebellar cortex became more active as the level of difficulty of a pitch discrimination task increased [90]. In addition, recent neuropsychological and neuroimaging studies have implicated left crus I in tasks involving biological motion perception [91, 92] (see also section by Drs. Sokolov and Pavlova, “Cerebellar Involvement in Biological Motion Processing (A.A. Sokolov and M.A. Pavlova)”), suggesting a role in higher-level visual processing.

Interestingly, there have also been incidental reports of cerebellar activity during tasks involving crossmodal matching [93–95]. For example, we observed that combined audiovisual motion detection led to increased activity bilaterally in cerebellar lobule VI and right lateral crus I, relative to unimodal visual and auditory motion tasks [93]. This is consistent with findings in monkeys that different sensory areas of the cerebral cortex converge on common areas within the neocerebellum [1]. Taken together, these results suggest that the cerebellar hemispheres play a role in the detection of intermodal invariant temporal–spatial features in concurrent streams of audio-visual information.

A prominent hypothesis is that the cerebellum aids information processing by making predictions, in the form of an “internal model” of sensory events [96]. An alternative account is that the cerebellum facilitates perception by monitoring and coordinating the acquisition of sensory information [97] (see the section by Dr. Bower, “Is the Cerebellum Sensory for Motor’s Sake, or Motor for Sensory’s Sake? (J.M. Bower)”). A third hypothesis is that the cerebellum functions as an internal timing device for both motor and perceptual processes, with different regions of the cerebellum thought to provide separate timing computations for different tasks [98] (see the section by Dr. Ivry, “Sensory Processing and the Cerebellum: Timing (R.B. Ivry)”). At present, there is no unequivocal support for any one of these models, and in fact, each can provide at least a partial account for many of the relevant findings.

In conclusion, while there is considerable evidence that the cerebellum contributes to auditory and visual sensory processes, its precise role is not yet well understood. We need more information about how the cerebellum interacts with visual and auditory networks, particularly in terms of the nature (inhibitory or excitatory) and directionality (feedback or feedforward) of these connections.

## Cerebellar Involvement in Biological Motion Processing (A.A. Sokolov and M.A. Pavlova)

**Fig. 4:**
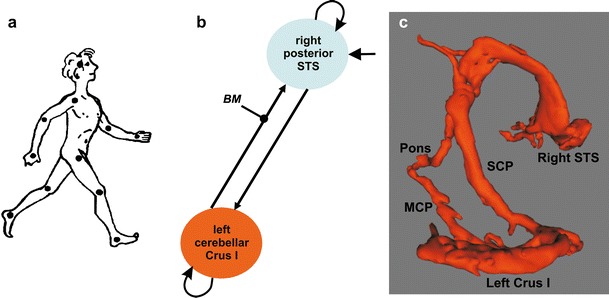
Loop between the cerebellum and superior temporal sulcus (STS) subserving biological motion perception. **a** Example of a point-light biological locomotion stimulus with 11 dots placed on the main joints of the walking human body. Outline added for illustrative purpose. From [246] Pion Ltd., London, www.envplan.com. **b** Dynamic causal modeling shows reciprocal effective communication between the right posterior STS and the left lateral cerebellar lobule crus I during visual processing of biological motion (BM) that modulates the back connection from the cerebellum to the STS. Adapted from [92], Copyright © 2011 Elsevier Inc., with permission of the publisher, Elsevier. **c** Three-dimensional representation of the structural loop pathway between the right STS and crus I, as revealed by diffusion tensor imaging (DTI). Fibers descending from the STS to the cerebellum pass through the pons and the middle cerebellar peduncle (MCP), while ascending fibers pass through the superior cerebellar peduncle (SCP) and the thalamus. From [37], copyright © The Author 2012. Published by Oxford University Press

Visual perception of bodily movements of others (for perception of self-motion, see the section by Dr Cullen, “The Cerebellum and Perception: The Role of the Cerebellum in Self-Motion Perception (K.E. Cullen)”) is essential for a wide range of daily life activities such as safe car driving, motor learning, imitation, social interaction, and non-verbal communication through body language [99]. Healthy adults and children easily recognize personality traits through actions of others, even if they are represented through a set of light dots placed on the main body joints, in “point-light biological motion displays” [100, 101] (see Fig. 4a). Neurophysiological and lesional research has revealed the core components of the cortical system underlying visual perception of body motion that includes areas in the frontal [102] and parietal [103–105] cortices, the fusiform gyrus and superior temporal sulcus (STS) [105–107], mainly in the right brain hemisphere [108]. Yet, our knowledge on engagement of brain structures outside the cerebral cortex is still rather limited.

Early positron emission tomography (PET) data suggest activation of the amygdala and left lateral cerebellum for point-light dance-like biological motion [103]. fMRI also indicates cerebellar activity during visual processing of body motion. However, the outcome is controversial, in particular, in respect to topography and lateralization. Right midline cerebellar response was found for a contrast of canonical against scrambled point-light actions when observers performed a one-back repetition task [109]. In a two-alternative forced choice (2AFC) discrimination task, bilateral activation in the cerebellar hemispheres was shown for canonical and scrambled point-light displays pooled together and contrasted against baseline, with specific activation of the left lateral cerebellar region QuP (posterior quadrangular lobule or lobule VI) when judging direction of biological motion [104].

Psychophysical data in patients with tumors to the left cerebellum showed that damage to the lateral lobules VIIB, VIIIA, and crus I and II substantially affects visual sensitivity to biological motion simultaneously camouflaged by additional moving dots (a spatially scrambled display containing the same characteristics as a canonical biological motion display (except for the spatial positions of the dots) served as a control for biological motion specificity in this series of studies) [91]. In contrast, sensitivity was not impaired in patients with lesions to the medial left cerebellum. In accord with lesional data, fMRI in a homogeneous group of healthy human adults indicated activation of the left lateral cerebellar lobules crus I and VIIB [92]. Convergent lesion and brain imaging findings provide reliable evidence in favor of involvement of the left lateral cerebellum in visual processing of human locomotion. Moreover, dynamic causal modeling demonstrated bidirectional task-related effective connectivity between the left lateral cerebellar lobule crus I and the right STS during body motion perception [92] (see Fig. 4b). The findings suggest that the cerebellum interacts with the cortical structure considered as a hub of the neural network subserving visual processing of biological motion [105–107]. This may account for effects of left lateral cerebellar lesions on visual tuning to biological motion [91].

While closed cerebellar loops with the frontal and parietal cortices are thought to underlie a variety of cognitive functions [110], direct communication between the temporal cortex and cerebellum during a visual perceptual task had not been previously shown. Neuroanatomical evidence in non-human primates points to direct projections from the STS to the pons [8, 9, 14, 111, 112] and from the pons to the cerebellum [27, 108]. However, there has been lacking evidence for a back connection from the cerebellum to the STS. Resting state fMRI analyses (see the section by Dr. Habas, “Resting-State Functional Connectivity Between Cerebellum and Sensory Systems (C. Habas)”) indicated possible functional connectivity between the cerebellum and temporal cortex [33–35]. A possibility for structural connection between the temporal cortex and cerebellum had been detected by diffusion tensor imaging (DTI) in non-human primates and humans [25]. By using high-resolution acquisition sequences and optimized processing, our latest DTI work indicates a bidirectional structural loop between regions in the left cerebellar lobule crus I and right STS that were functionally defined during visual processing of biological motion [37] (see Fig. 4c).

In neuropsychiatric conditions such as schizophrenia or autistic spectrum disorders (ASD), impaired biological motion processing [99, 113, 114] and altered cerebro-cerebellar connectivity [115, 116] represent two major characteristics. Yet the relationship between these characteristics has not been experimentally investigated. Reciprocal loops between the cerebellum and STS in visual processing of body motion may account for lower STS response to biological motion in children with ASD [117] and help to explain how social deficits relate to disintegrity of the left superior cerebellar peduncle [118] hosting the back connection from the cerebellum to the STS [37]. Cerebellar involvement in biological motion processing instigates further research on social brain networks in neuropsychiatric conditions.

In a nutshell, the left lateral cerebellum appears to be strongly involved in visual processing of biological motion [91, 92]. This engagement occurs likely through direct reciprocal communication with the right STS [37, 92], a keystone of brain networks for body motion processing and visual social cognition [99, 105–107]. Both specificity of deficits in patients with cerebellar lesions and network topography in healthy adults suggest that cerebellar engagement in biological motion processing and action observation goes beyond a general role of the cerebellum in visual motion processing [86, 119; see also the section by Drs. Baumann and Mattingley, “The Role of the Cerebellum in Visual and Auditory Processing (O. Baumann and J.B. Mattingley)”]. Recent data indicate a remarkable potential for recovery of visual body motion processing following neurosurgical left cerebellar lesion removal and suggest that reorganization in the cerebellum may trigger topographic shifts in the communicating superior temporal areas [120]. The exact function of the cerebellum within the circuitry for perception of biological motion needs further clarification. Engagement of both the left cerebellum and right STS has been reported in emotion recognition through body motion [121], detection of social interaction and animacy attribution in Heider-and-Simmel movies depicting geometric shapes [122–124], imitation [125], and audiovisual integration ([93]; see section of Drs. Baumann and Mattingley, “The Role of the Cerebellum in Visual and Auditory Processing (O. Baumann and J.B. Mattingley)”). Effective connectivity between the cerebellum and STS during animacy attribution has recently been demonstrated [124]. Further studies are needed to clarify whether and how communication between the cerebellum and STS might underlie other social cognitive functions, and to address compensatory potential in congenital, degenerative, and focal cerebellar affections.

## The Cerebellum and Perception: The Role of the Cerebellum in Self-Motion Perception (K.E. Cullen)

The cognitive representation of self-motion is vital to our everyday activities. For instance, walking down a busy city street requires an accurate estimate of our own motion relative to objects in the surrounding complex, three-dimensional environment. Self-motion requires the integration of sensory information from multiple systems including vestibular (head motion), visual (optic flow), proprioceptive, and somatosensory (body motion), as well as efference copy motor command signals (reviewed in [126]).

There is strong evidence that the cerebellum, and, in particular, the vestibulo-cerebellum, makes vital contributions to self-motion perception. First, it has long been known that lesions of the nodulus and uvula (Larsell’s lobules X and IX) alter the temporal and three-dimensional spatial processing of vestibular information (reviewed in [127]). More recently, it has been further shown that visually induced illusions of self-motion preferentially activate these same lobules [128, 129] and that self-motion perception is diminished in patients with midline lesions impacting these regions [130, 131]. Thus, the vestibulo-cerebellum is thought to be required for computing the internal representation of self-motion.

Recent electrophysiological analyses of the vestibulo-cerebellum and vestibular sensory pathway of monkeys have provided important insights into the specific neural computations underlying the integration of multimodal information required for self-motion perception.

First, to generate an accurate perception of our motion relative to the world, the brain must continuously account for the omnipresent force of gravity. The brain constructs internal models of the world’s physical laws to dissociate tilt from translation by combining inputs from the vestibular otoliths (which detect linear motion for both movements) with inputs from semicircular canals (which detect rotational motion, and thus only respond to tilts) [132]. Consistent with this proposal, single nodulus-uvula neurons create an internal model that accounts for the physics of our world. Notably, neuronal responses to rotations are modulated as a function of head orientation relative to gravity (reviewed in [127]) and different subclasses of Purkinje cells encode head translation versus tilt [133]. This representation of translation could potentially be combined with the visual and proprioceptive input to provide an estimate of heading direction that is based on information from multiple sensory systems.

Second, to perceive body motion independently of head motion, the brain must compare vestibular and neck-related inputs. Direct evidence for this computation has been revealed in the output of the cerebellum, at the level of the neurons in the most medial of the deep cerebellar nuclei (i.e., fastigial), which comprises two distinct populations of neurons. One neuronal population responds to both externally applied vestibular and neck-proprioceptive stimulation, and encodes body-in-space motion. The other neuronal population only responds to externally applied vestibular inputs and encodes head-in-space motion [134]. Notably, the convergence of vestibular and proprioceptive inputs in body coding cerebellar neurons is non-linear [134] and likely underlies the transformation of vestibular signals from a head to a body reference frame in the deep cerebellar nucleus [135, 136].

Finally, to ensure perceptual stability in everyday life, our brains must continually distinguish between self-motion that is the result of our own (active) movements versus externally applied (passive) motion. Theoretically, the computation of passive motion requires a comparison between an internal estimate of the sensory consequences of active self-motion (i.e., forward model) and the actual sensory feedback (reviewed in [126]). Cerebellar output neurons dynamically encode this difference during self-motion; fastigial neurons are insensitive to active motion and encode an explicit representation of passively applied self-motion [137]. Specifically, the two distinct fastigial nucleus populations (described in the paragraph above) selectively and dynamically encode passive head and body motion relative to space. Moreover, our evidence to date suggests that this cerebellar-dependent mechanism uses an internal model of the expected sensory consequences of active head motion to selectively cancel responses to active motion.

**Fig. 5:**
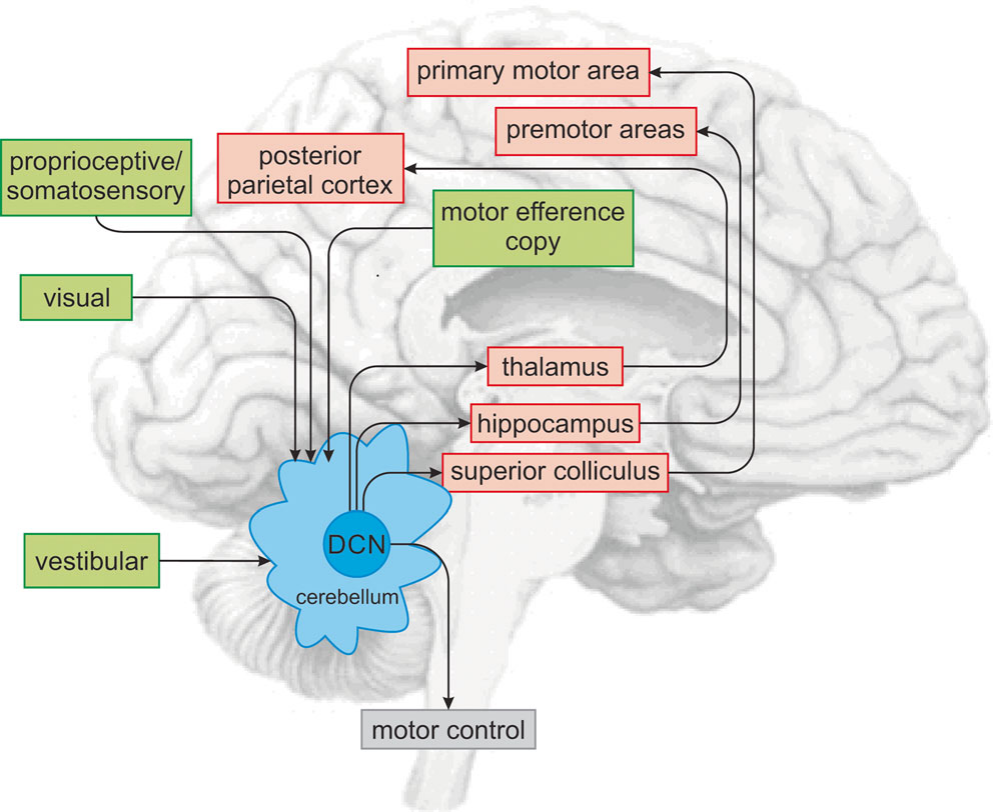
The cerebellum integrates sensory input (*green boxes*) from multiple systems including: (1) the vestibular, (2) visual, (3) proprioceptive and somatosensory, as well as from (4) motor efference copy signals. Cerebellar output neurons send ascending projections to the thalamus, hippocampus, and superior colliculus, which in turn connect the cerebellum to numerous cortical regions (*red boxes*) that mediate spatial navigation and voluntary motor control

In summary, computations in the vestibulo-cerebellum underlie the transformation of input signals into representations that are essential for self-motion perception (Fig. 5). Interestingly, these same cerebellar-dependent computations likely also contribute to mapping spatial representation in the hippocampus (Fig. 5, ascending pathway in red). Notably, ‘place cell’ tuning is impaired in mutant mice with cerebellar function deficits [138]. The cerebellum likely shapes the directional tuning of place cells via indirect projections from the deep cerebellar nuclei. Moreover, ascending projections terminate in regions of the thalamus [139] known to terminate in parietal cortex, a region that is vital for spatial navigation, as well as motor and premotor cortex [140]. Future work in monkeys and mice using both passive and active motion are needed to fully understand the impact of the cerebellum on how the hippocampus and cortex shape spatial navigation.

## Pain and the Cerebellum (R.J. Borra and E.A. Moulton)

The cerebellum is one of the most consistently responsive brain structures to painful stimuli [141]. While our classical understanding of this structure suggests that it is involved in the motor response to pain, contemporary thinking indicates that it may have a more direct role in the processing of pain. The perception of pain itself is a complex subjective experience that incorporates sensory, affective, and cognitive components. Though neuroimaging studies indicate that the cerebellum responds to noxious stimuli, its functional relevance in relation to these different dimensions is only starting to gain attention.

### Ascending Nociceptive Input to the Cerebellum

Well-controlled studies of pain often use acute experimental stimuli to activate nociceptive pathways, the physiological processes underlying pain perception. Nociceptors are primary afferents that respond to high threshold mechanical and heat stimuli, and can also respond to chemical stimulation, such as during inflammation. Two major categories of nociceptive afferents have been classified: A-delta and C-fiber nociceptors. A-delta nociceptors are thinly myelinated and fast conducting (>2 m/s), while C-fiber nociceptors are unmyelinated and slower conducting (<2 m/s). Electrophysiological studies in rodents and cats indicate that stimulation of cutaneous and visceral nociceptors, in the form of A-delta and/or C-fiber primary afferents, can activate and modulate Purkinje cell activity in the cerebellum [141, 142]. At least two possible nociceptive spinocerebellar pathways have been proposed: (1) a spino-olivocerebellar pathway that conveys A-delta and C-fiber nociceptive afferent input to Purkinje cells in the cerebellar anterior lobe ipsilateral to stimulation [143] and (2) a spino-pontocerebellar pathway conveying C-fiber nociceptive input to Purkinje cells in the cerebellar vermis [144]. Details regarding these putative pathways have been vastly understudied.

### Descending Cortical and Subcortical Input to the Cerebellum

In addition to afferent input, the cerebellum receives input from brain areas associated with nociceptive processing, including cognition, affect, and motor function [141]. Our current understanding of the neural basis of pain and its modulation includes the somatosensory cortices, periaqueductal gray, anterior cingulate cortex, dorsolateral prefrontal cortices, basal ganglia, hippocampus, hypothalamus, and the amygdala [145], all of which have connectivity with the cerebellum [1, 96]. With the cerebellum receiving both descending information from other brain areas and ascending nociceptive information from the spinal cord, the structure is ideally positioned to influence, or to be influenced by, the processing of pain.

### Neuroimaging Responses to Pain in the Cerebellum

A meta-analysis of 47 neuroimaging studies featuring experimental pain revealed specifically localized responses within the cerebellar vermis and bilaterally in the posterior hemispheres [141]. The spatial extent of vermal activation spanned across vermal lobules III, IV, and V, while the bilateral hemispheric activation spanned from hemispheric lobule VI to crus I. Using the same method of meta-analysis, a similar pattern of activation was observed across 16 neuroimaging studies featuring pathological pain, in the form of spontaneous pain or aggravation of a clinical condition.

Though pain neuroimaging studies are not typically designed to evaluate the physiological significance of cerebellar responses, a few notable studies have focused in on this structure in the context of pain. Helmchen and colleagues used fMRI to find that activation in hemispheric lobule VI and in the anterior vermis varied with subject reports of pain intensity, though only when the stimuli were self-administered [146]. The authors suggested that these cerebellar regions could reflect pain perception and are involved in signaling the expected sensory consequences of pain. In another study, fMRI of trigeminal neuropathic pain elicited by brushing and heat showed responses in crus I, crus II, and lobule VIIB that were not evoked by the non-painful control stimuli [147].

Recent neuroimaging evidence suggests that certain cerebellar responses during pain may reflect multi-modal aversive processing. An fMRI study found that noxious heat and the passive viewing of unpleasant pictures activated overlapping regions of the cerebellum: hemispheric lobules VI, VIIB, and crus I [148]. Further analysis revealed that these areas of functional overlap were significantly inversely correlated with activation in the anterior hypothalamus, subgenual anterior cingulate cortex, and the parahippocampal gyrus. These findings suggest that responses in these cerebellar regions are not specific to pain processing but appear to apply to other aversive sensory and affective experiences as well [149]. However, other functions related to pain aside from aversion may also be processed in the cerebellum, as areas that responded to noxious heat and not to aversive pictures were also identified including crus II. Further study is required to determine the functional topography of the cerebellum as it relates to pain and its different sensory, affective, and cognitive components.

## Sensory Processing and the Cerebellum: Timing (R.B. Ivry)

**Fig. 6:**
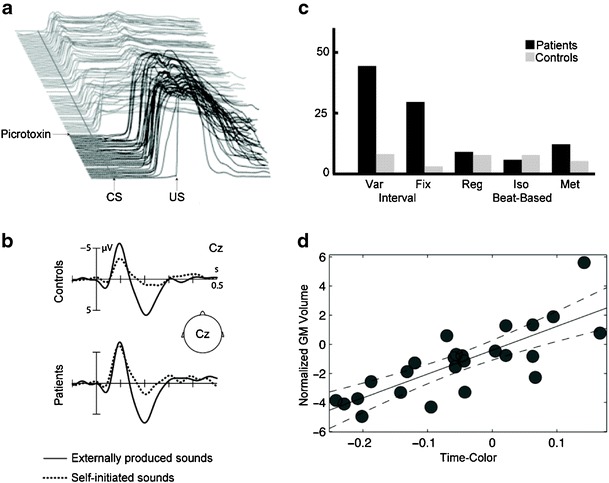
Cerebellum and sensory timing. **a** Adaptive timing of conditioned eye blink response is abolished following infusion of picrotoxin, an agent that disrupts input from cerebellar cortex to deep cerebellar nuclei. Courtesy of Michael Mauk. **b** Patients with focal cerebellar lesions fail to show attenuated ERP response to self-generated sounds compared with externally produced sounds. Adapted from [156]. **c** Patients with cerebellar degeneration (SCA6) exhibit selective deficit on time perception tasks that require interval timing (Var, Fix) while spared performance on tasks that require beat-based timing (Reg, Iso, Met). Adapted from [165]. **d** Cerebellar grey matter volume is correlated with perceptual acuity on time discrimination task, relative to a color discrimination task. Adapted from [173]

Movement dynamically incorporates sensory information and anticipates the sensory consequences of the action (see also the section by Dr. Bower, “Is the Cerebellum Sensory for Motor’s Sake, or Motor for Sensory’s Sake? (J.M. Bower)”). While this is a general feature of motor control, there is consensus of a cerebellar dependency on tasks that impose precise temporal constraints. A prominent feature of cerebellar ataxia is the loss of the fine temporal patterning that is characteristic of skilled movement. Experimentally, eyeblink conditioning has proven to be an exquisite model system for studying cerebellar-dependent timing [150, 151]. This form of learning is only adaptive if the animal is able to represent the temporal relationship between two sensory events, the conditioned and unconditioned stimuli. Importantly, the conditioned response persists following lesions of the cerebellar cortex but loses its adaptive timing [152] (see Fig. 6a). Sensory timing as a constraint on motor control is also evident in many tasks involving volitional movements. To intercept a moving object, the movement has to anticipate the trajectory of the object. Patients with cerebellar lesions have great difficulty with such tasks [153]. Mice lacking genes associated with cerebellar-dependent plasticity are selectively impaired in an operant task that requires using precise sensory timing to restrict movement latencies [154].

The preceding examples highlight a critical cerebellar role in using sensory information to time movement. The reverse situation, where movement is used to anticipate and modulate sensory information, is also cerebellar dependent, at least when the events are of a limited temporal extent. We have general consensus that the cerebellum uses a forward model to generate a prediction of the expected sensory consequences of an action [155]. Kotz and colleagues [156] provide a particularly compelling EEG example. The early N100 response evoked by an auditory stimulus is markedly attenuated when the tones are triggered by a volitional action compared with when the tones are externally triggered. This attenuation is essentially absent in patients with focal cerebellar lesions of the left or right hemisphere, with the sensory response similar for self-triggered and externally triggered actions (see Fig. 6b).

Forward models, as a form of prediction, have been employed to describe brain function more generally [157]. A challenge is to specify the conditions that distinguish cerebellar-dependent and cerebellar-independent forward models. One possibility is that, as with classical conditioning, the cerebellar domain is defined by temporal constraints, situations in which the predictions require some form of precise temporal representation. In one oft-cited example, the tickling sensation from self-generated movements becomes more intense when delays are introduced between the action and the somatosensory stimulation [158]. Similarly, learning rates are dramatically reduced with delayed feedback during visuomotor adaptation [159].

The strongest evidence for a critical role of the cerebellum in sensory timing comes from tasks that do not entail overt movement (see also the section by Drs. Baumann and Mattingley, “The Role of the Cerebellum in Visual and Auditory Processing (O. Baumann and J.B. Mattingley)”). Research here falls into three general domains. First are tasks examining how the cerebellum responds to temporal regularities, or perhaps more telling, violations of temporal expectancies. Tesche [160] compared evoked MEG responses to periodic (predictable) tactile stimuli or epochs in which the stimulus was withheld (prediction violations). Whereas the evoked response in somatosensory cortex was stimulus-locked and independent of predictability, the cerebellar response was anticipatory, leading the expected onset of the stimulus. Moreover, it was markedly larger following a violation, consistent with the idea that the cerebellum was sensitive to temporal prediction violations. Further support for this idea comes from fMRI work showing larger cerebellar activation to visual stimuli with unpredictable timing (e.g., [161]) as well as a study in which an early ERP signal to deviant auditory stimuli was found to be abnormal in patients with cerebellar degeneration [162].

The second domain involves studies of velocity perception. Cellular activity in the posterior cerebellum is sensitive to stimulus motion (see sections by Dr. Cullen on “The Cerebellum and Perception: The Role of the Cerebellum in Self-Motion Perception (K.E. Cullen)”, and Drs. Sokolov and Pavlova, “Cerebellar Involvement in Biological Motion Processing (A.A. Sokolov and M.A. Pavlova)”). It is possible that these signals are related to preparation of potential eye or body movements. However, a causal contribution to perception comes from psychophysical studies showing that patients with cerebellar pathology are impaired in visual motion discrimination [86, 163]. Moreover, the cerebellar contribution appears to be most critical when the motion perception task requires time-based judgments. O’Reilly [164] used a task in which a moving stimulus disappeared behind an occluder. When the stimulus reappeared, the participant had to judge if there had been a deviation in direction (spatial) or speed (temporal). The cerebellar BOLD response was larger in the latter compared with the former. Converging evidence comes from a study showing that patients with cerebellar pathology are impaired in adapting to velocity perturbations in this task [58].

Third, and perhaps most direct, are studies of duration discrimination. Ivry and Keele [88] provided the first evidence of a “pure” sensory timing deficit in patients with cerebellar pathology. The patients were impaired in judging the duration of an auditory stimulus but showed normal performance in judging stimulus loudness. This finding has been confirmed in various studies over the past 25 years, including one study in which testing was restricted to a large group of patients with SCA6, a condition in which the pathology is relatively restricted to the cerebellar cortex [165] (see Fig. 6c), and studies with healthy individuals in which cerebellar function has been transiently disrupted by TMS [166, 167]. There is general consensus that cerebellar contributions to sensory (and motor) timing are most pronounced with relatively short intervals (less than 1 s) and in the representation of intervals (either absolute or relative as in state estimation models) rather than more complex temporal relationships (e.g., rhythm). The few negative results on duration discrimination are also informative: They have involved patients with unilateral lesions [168, 169], suggesting that a single intact cerebellar hemisphere may be sufficient to support sensory timing [170]. The functional neuroimaging literature on duration perception has proven more difficult to decipher [171], especially since many studies do not provide adequate coverage of the cerebellum. Interestingly, three recent structural MRI studies report a positive correlation between measures of cerebellum volume and temporal acuity in healthy individuals [172–174] (see Fig. 6d).

This is not to say there is consensus for the uniqueness of the cerebellum in sensory timing. Indeed, there is consensus that the cerebellum is not the sole structure capable of representing temporal information. The challenge remains to develop more analytic tasks and models that provide better specification of the various operations required in tasks that require precise temporal processing. Nonetheless, the cerebellar timing hypothesis [98] has proven to be of considerable utility for exploring the function, structure, and physiological of the cerebellum in motor control and beyond.

## The Cerebellum in Predicting Perceptual Events (M. Leggio and M. Molinari)

Perception can be considered the result of interactions in time between a dynamic mind and a dynamic world. To achieve mind-world synchronization, our perceptual systems must constantly tune themselves to an ever-changing environment. Perceptual tuning, like the sensorimotor tuning that is needed for smooth movement control, can be obtained only if prediction capabilities are embedded in the process [175]. Moreover, predictive processing represents a fundamental principle of neural computations in the brain [176].

Many groups have attempted to identify the neural bases of foresight, and despite considerable ongoing debate, a consensus exists on the importance of the cerebellum in prediction [177]. To make the matter even more interesting for cerebellar scientists, data are accumulating on the significance of the cerebellum for sensory processing and in optimizing perception [58]. Perceptual optimization and prediction of incoming sensory information have been suggested to be effected by sequence processing in cerebellar circuits [58, 178, 179].

Using magnetoencephalographic recordings, Tesche and Karhu [160] demonstrated that cerebellar activity is enhanced after an unpredictable omission is inserted into a regular train of somatosensory stimuli. As a result, no activity is present in the parietal cortex, whereas a notable response develops in the cerebellum. Consequently, it can be argued that the cerebellum detects the absence of a somatosensory stimulus to a greater extent than its presence. This response to the absence of a stimulus can be understood only as an indication that something that is expected does not appear [180]. If a sensory pattern is recognized, it is possible to predict the sequence of events and consequently anticipate each one [181]. Thus, in predicting incoming sensory information, the cerebellum governs the detection of the absence of an expected stimulus and the appearance of an unexpected stimulus.

**Fig. 7:**
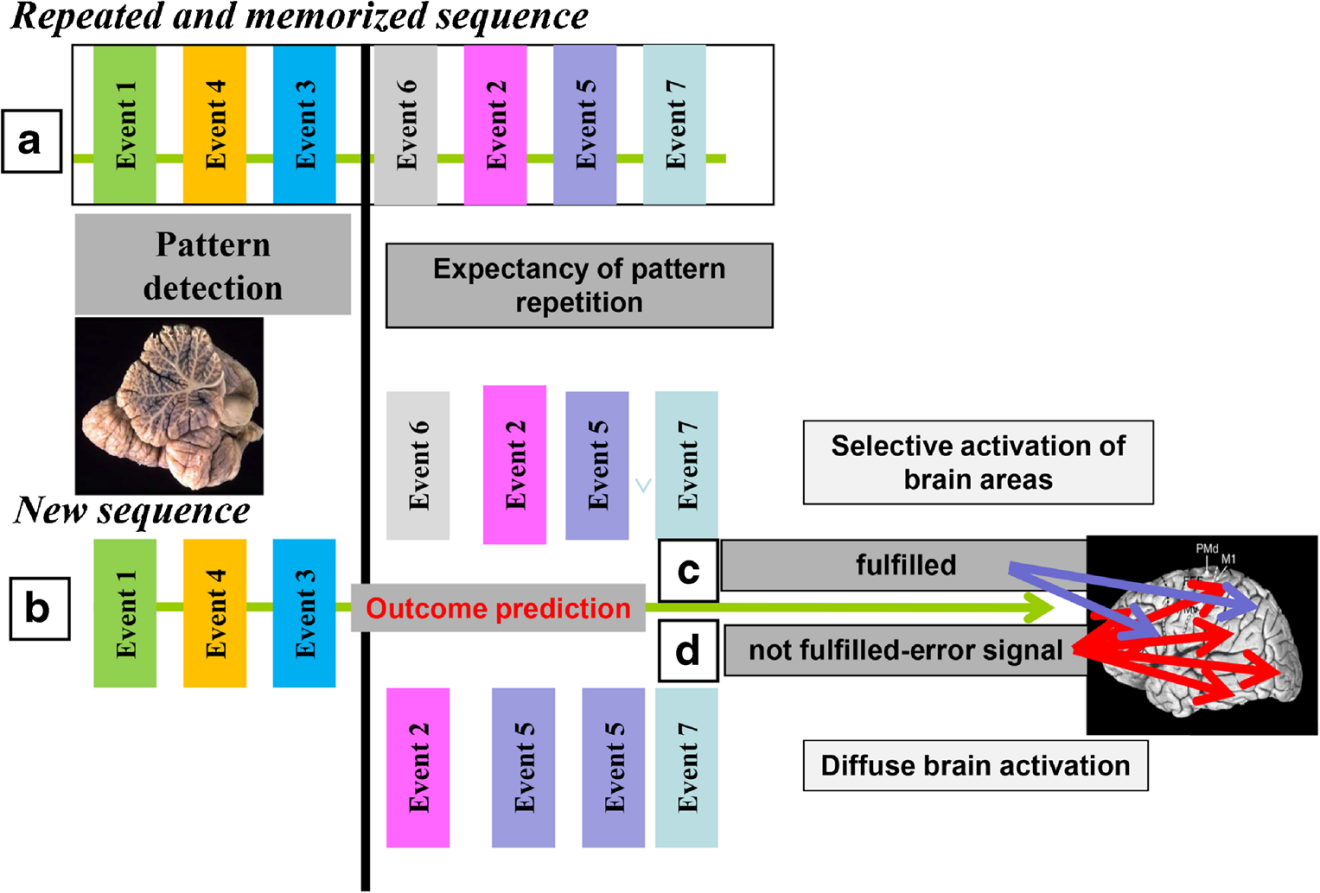
Sequence detection model of prediction. If sensory events appear in a fixed sequence repeatedly in a short time, the sensory sequence is implicitly memorized **a** which allows cerebellar circuits to compute a prediction for forthcoming perceptual events **b**. If the prediction holds **c**, a signal is sent to the cerebral cortex to alert selective brain areas, which become activated prior to the realized event and are thus better suited to process the incoming stimulus. If the prediction fails **d**, an alert signal is sent, and brain activation is more widespread, accelerating the processing of salient sensory information by the changing events and attuning the behavioral response to the new environment

Mismatch negativity (MMN) studies in subjects with cerebellar damage in the somatosensory [182] or auditory [162] domain have confirmed this hypothesis. MMN is believed to be generated by an automatic cortical change-detection process that is activated by differences between current and prior inputs. When the MMN protocol is applied to subjects with cerebellar lesions, the MMN response is absent or abnormal. Per the long-standing model in which the cerebellum acts as a comparator [183], it has been proposed that, in the cerebellum, actual input and preceding stimuli are compared, and discordances are identified. If the incoming stimulus corresponds to the predicted stimulus, cerebellar output is minimal; if a discrepancy–error signal is detected, the activity in the cerebellum increases and a large area of the cerebral cortex is alerted by enhancing its excitability (Fig. 7).

We developed a “sequence detection model” to describe the operational mode of cerebellar processing not only in somatosensory [182], but also in visuospatial [184] and cognitive domains [185]. Cerebellar patients were impaired specifically in the recognition of spatial sequences when tested on a visuospatial serial reaction time task [184]. Results of visuospatial tests demonstrated that subjects with cerebellar damage were impaired specifically with regard to sequence recognition, even to a greater extent than sequence execution [184]. Furthermore, by forcing the declarative knowledge of the spatial order, it was possible to improve performance significantly. Similar findings have been reported by several groups [186–191], supporting cerebellar function in extracting sequential order information from incoming sensory information [184].

Subjects with cerebellar damage also develop impairments in cognitive sequencing [192]. We analyzed prediction ability in patients with cerebellar damage who performed a cognitive task in which predictability was based primarily on abstract/spatial, behavioral/visual, or behavioral/linguistic sequence information [192]; in this task, sets of cartoon-like drawings that reproduced behavioral sequences were to be placed in the correct order. The patients were impaired in sequencing events in all domains, developing domain-related specificity, based on the side of the cerebellar lesion. Thus, although no specific sequencing localization can be identified, sequencing processing can be found in the different cerebellar functional domains. This impairment suggests difficulties in perceiving the depicted behavior correctly. This evidence is consistent with difficulties that are encountered in tuning behavior and the environment correctly not only after cerebellar damage [19], but also in behavioral pathologies, such as autism and schizophrenia, disorders that have been linked to cerebellar abnormality [193, 194].

The hypothesis that pattern detection and prediction represent a specific role in cerebellar function in perception is appealing, and compelling data from various sources support the sequence detection model of impaired cerebellar perception. Furthermore, the perceptual deficits that are observed in schizophrenia [195, 196] and autism [197, 198] resemble cerebellar dysfunctions. Notably, cerebellar pathogenic mechanisms have been hypothesized to mediate schizophrenia [199] and autism [200], and the existence of cerebellar-like sequence detection deficits [201, 202] is additional support for the cerebellar pathogenic theories of these diseases.

## Is the Cerebellum Sensory for Motor’s Sake, or Motor for Sensory’s Sake? (J.M. Bower)

This title is the same as a paper published more than 15 years ago describing our hypothesis that the cerebellum controls the acquisition of sensory data [203], an idea first proposed even 10 years earlier in a paper exploring the spatial structure of the extensive peri-oral tactile representations in the cerebellum of rats:

“… we suggest that tactile regions of the cerebellum are involved in controlling the movements specifically associated with active tactile exploration …to coordinate the use of sensory structures so that the highest quality sensory information is being obtained by the rest of the nervous system during the exploratory process. By monitoring the acquisition of sensory information and adjusting motor performance accordingly, cerebellar circuits would be expected to substantially improve the efficiency of sensory processing by the rest of the nervous system.” (p. 776, [204])

### Evidence in Support

While a model-based re-analysis of cerebellar cortical networks also supports this hypothesis [205], this review will focus on supporting experimental results from more behavioral including human studies. First in a series of imaging experiments, we demonstrated, as the hypothesis predicts, that activity in the human cerebellum [51, 206] and related structures [207] is substantially greater when fingers are used in a tactile discrimination task. A meta-analysis of neuroimaging data then generalized this result to the auditory system, suggesting larger and more spatially extensive activations during discriminative auditory tasks [208], a result subsequently confirmed using PET [90]. Importantly, the PET study also supported a further important prediction of the sensory acquisition hypothesis, namely that cerebellar activity should increase with task difficulty, i.e., when better control of the quality of sensory data is likely more important [97]. A similar result has been reported independently in a combined human visual and auditory imaging study [89].

While human imaging data can be suggestive of brain function, an important test of any functional hypothesis is its ability to predict behavioral results. In this case, it has long been a central tenet of cerebellar descriptions that the structure has no influence on sensory perception [209]. However, because sensory perception is based on the quality of sensory data, we predicted that impairment of cerebellar function should have sensory perceptive consequences [203]. Consistent with this prediction, we have shown that humans with cerebellar degenerative disease have significantly poorer thresholds for pitch discrimination [57]. Other studies in audition [86], somatosensation [210, 211], proprioception, and vision [212] have now also demonstrated cerebellar-related primary sensory deficits, which have also been reported using higher order tasks like speech [213], motion detection [214], analysis of temporal sequences [178], as well as the general perception of time [167, 215].

Finally, while human psychophysical and imaging studies, properly designed, can test functional hypotheses, linking these hypotheses to actual physical computational mechanisms still requires the use of animal models [205]. While the large majority of animal studies exploring the functional significance of the extensive sensory projections to the cerebellum continue to frame the results in the context of traditional motor control theories [216], a recent behavioral study in rats has demonstrated that optogenetic stimulation of the cerebellum specifically disrupts the use of the whiskers during active touch [217]. These authors specifically conclude that their results support a role of the cerebellum in the “optimization of sensory data acquisition” (p. 6, [217]).

### Implication for Theories of Cerebellar Sensory Function

With growing evidence that the cerebellum plays some role in sensory function, it is time to fully reconsider cerebellar function from a sensory point of view:

1. *Re-interpreting cerebellar involvement in motor control.* It has been known for more than 150 years that lesions of the cerebellum disrupt movement [218, 219] with the majority of cerebellar theories accordingly focused on mechanisms of direct motor control [220]. In contrast, the sensory data acquisition hypothesis proposes that cerebellar effects on movement are an indirect consequence of disrupting the sensory data on which motor behavior depends [97]. This prediction is consistent with recent evidence that cerebellar patients have difficulty discriminating proprioceptive stimuli [210] and that a significant component of cerebellar ataxia results from the inability of patients to perceive environmental instabilities [221]. For this reason, it is critically important that motor-related studies, perhaps especially those involving purported motor learning [222], control for cerebellar effects on primary sensory data.
2. *Removing the legacy of cerebellar motor control theories.* At present, most explanations for cerebellar involvement in non-motor-related behaviors assume that evolution has adopted cerebellar motor control computational mechanisms to non-motor tasks [58, 73, 92, 211, 220, 223–226], including, for example, a presumed general role for the cerebellum in timing not only of muscle activations during movement but also of sensory perception [98, 227]. While our analysis of cerebellar cortical circuitry questions the circuitry-based evidence for the original timing hypothesis [205], we do expect that any disruption in sensory data acquisition control may very well be particularly apparent with tasks involving precise timing (see the section by Dr. Ivry, “Sensory Processing and the Cerebellum: Timing (R.B. Ivry)”). This is not, however, because the cerebellum itself implements a timing function, but instead because sensory information is temporally coded at the neuronal level [228], and therefore experimental manipulations of expected timing relationships in presented stimuli are likely to evoke stronger cerebellar effects.
3. *The cerebellum is invoked in proportion to the need for sensory vigilance.* Another important prediction of our hypothesis is that cerebellar involvement will scale as better controlled sensory data are required [90], making it important to evaluate task difficulty when considering cerebellar-related sensory effects [58, 229, 230]. Interestingly, numerous cerebellar studies already employ masking sensory noise to evoke larger cerebellar responses [89, 92] or reveal behavioral deficits [163]. Overcoming the consequences of sensory noise either applied externally or self-generated [231] we predict will especially increase requirements for cerebellar control. This effect also confounds the interpretation of sensory stimuli like pain [141], which on their own increase subject vigilance [148, 232], as well as studies of mechanisms like attention [233–235].
4. *The cerebellum is a support structure.* Perhaps the most important implication of the sensory hypothesis is that the cerebellum performs a more internal than external function. Instead of itself contributing directly to sensory perception, the influence of the cerebellum is predicted to be indirect, facilitating the computational efficiency of the rest of the brain, including cerebral cortex [163]. To quote again from 25 years ago:
  “It has been largely accepted that the flocculus of the cerebellum is involved in adjusting the gain of the vestibulo-occular reflex to assure a minimal slip of images on the retina during head movement [236, 237]. Psychophysical experiments demonstrate that more than 3°/sec of retinal slip starts to significantly degrade visual acuity and thus the ability of the visual system to process sensory information [238]. Thus the proposed role of the cerebellum, in VOR control, is to assure that the highest possible quality of visual information is provided to the visual system. In principle, this role is analogous to the role we are suggesting for lateral tactile regions of the cerebellar cortex.” (p. 776, [204]).
  For sensory systems like vision, audition, olfaction, and somatosensation, which in humans involve the largest part of the cerebellum, the ‘support system’ status of cerebellum also suggests a different interpretation of the important relationship between the cerebellum and the cerebral cortex. While in the traditional motor-control context, the influence of the cerebral cortex on the cerebellum is generally described as implementing a kind of forward model to (quoting Dr Ivry in this article) “generate a prediction of the expected sensory consequences of an action” (see also the section by Dr. Paulin, “Evolutionary Perspectives on Cerebellar Function (M.G. Paulin)”), in our view, the influence of the cerebral cortex on the cerebellum provides contextual information related to the expected use of the sensory data by cerebral cortex. We don’t think that such a function explicitly involves a ‘prediction’ as much as it does a continuous stream of contextual information. In fact, although again beyond the scope of the current commentary, our analysis of cerebellar cortex suggests that its circuitry specifically places information arising from particular sensory receptors (e.g., the upper lip) in the context of other sensory surfaces involved at the same time in sensory data acquisition (e.g., the lower lip). We have proposed that cerebellar output (through direct projections to the midbrain and brain stem motor centers as well as potentially through motor regions of cerebral cortex) then makes subtle relative adjustments in the position of tactile sensory surfaces to optimize the information content. A recent analysis of the influence of the cerebellum on whisking in rats supports this prediction [217]. Similarly, we have proposed that the cerebellum also likely modulates the cochlear outer hair cells during auditory data acquisition. In fact, we have suggested that the cerebellum plays the same role for all sensory systems.
5. *Implications for human disease.* Finally, the most exciting application of this sensory focused hypothesis may be to human health and disease. Although understudied, it has been known for more than 150 years that motor control can recover after cerebellar cortical lesions [239, 240] an effect also now demonstrated for presumed ‘cognitive’ function [241–243]. The sensory hypothesis attributed this recovery to the eventual adaptation of the rest of the brain to less well-controlled sensory data [203]. Evidence has also been growing that the cerebellum plays a role in autism spectrum disorders (ASD), although there is no consensus for the mechanism [244]. In the context of our hypothesis, the relationship is quite direct, with ASD seen as a behavioral adaptation to a general and overwhelming lack of control over the process of sensory data acquisition. From this perspective, therapies that focus on repetitive behaviors in highly controlled sensory environments with specific emphasis on sensory integration [245] would, we suggest, establish sensory conditions making it easier for the brain to learn to compensate for the lack of stable sensory data. It may even be worth considering whether the apparent increasing incidence of ASD could be attributable to sensory over-stimulation of children before the late developing cerebellum is fully functional.
  In summary, there is no question that the evidence is growing for some kind of cerebellar involvement in mechanisms of sensory function. However, instead of assuming a direct role in these mechanisms borrowing traditional cerebellar theories designed to explain motor control, in our view, this new evidence should instead call into question the historical view of the cerebellum as primarily a motor control device.

## Summary and Conclusions

The aim of this consensus paper is to capture the range of experimental approaches and theoretical models that have contributed to our current understanding of the influence of the cerebellum on perceptual processes. Contributions from fourteen experts, spanning a range of methodological approaches and with different theoretical views, have been brought together to provide an up-to-date snapshot of thinking on this topic.

The outcome of this project indicates that no single, coherent model has yet emerged regarding the mechanisms by which the cerebellum may influence perception. Nonetheless, it is important to assemble the empirical data, showing the association of the cerebellum with a wide range of perceptual systems including those related to vision, audition, touch, proprioception, self-motion perception, and nociception. The possible anatomical and physiological underpinnings of this broad influence was reviewed by Dr. Schmahmann, documenting significant cerebellar connection with sensory, as well as associative and paralimbic, areas of the cerebrum. These findings are corroborated by human neuroimaging studies, which show that fMRI resting-state signals in the cerebellum correlate significantly with those in visual and auditory cortices in the cerebrum (see the section by Dr. Habas, “Resting-State Functional Connectivity Between Cerebellum and Sensory Systems (C. Habas)”). Second, a number of the commentators described clinical studies that show how cerebellar lesions can lead to deficits in a diverse set of perceptual tasks, including visual motion perception, auditory pitch perception, self-motion perception, biological motion perception of others, time perception, and the recognition of perceptual sequences (see sections by Drs. Baumann and Mattingley, “The Role of the Cerebellum in Visual and Auditory Processing (O. Baumann and J.B. Mattingley)”; Drs. Pavlova and Sokolov, “Cerebellar Involvement in Biological Motion Processing (A.A. Sokolov and M.A. Pavlova)”; Dr. Cullen, “The Cerebellum and Perception: The Role of the Cerebellum in Self-Motion Perception (K.E. Cullen)”; Dr. Ivry, “Sensory Processing and the Cerebellum: Timing (R.B. Ivry)”; Drs. Leggio and Molinari, “The Cerebellum in Predicting Perceptual Events (M. Leggio and M. Molinari)”; and Dr. Bower, “Is the Cerebellum Sensory for Motor’s Sake, or Motor for Sensory’s Sake? (J.M. Bower)”). Third, human neuroimaging studies have consistently shown reliable cerebellar activation during performance of a range of perceptual tasks, independent of any motor-related activity of observers (see sections by Drs. Baumann and Mattingley, “The Role of the Cerebellum in Visual and Auditory Processing (O. Baumann and J.B. Mattingley)”; Drs. Pavlova and Sokolov, “Cerebellar Involvement in Biological Motion Processing (A.A. Sokolov and M.A. Pavlova)”; Drs. Borra and Moulton, “Pain and the Cerebellum (R.J. Borra and E.A. Moulton)”; and Dr. Bower, “Is the Cerebellum Sensory for Motor’s Sake, or Motor for Sensory’s Sake? (J.M. Bower)”).

In summary, it seems the answer to the question of whether the cerebellum plays a role in perception is unequivocally affirmative. What remains to be determined is precisely how the cerebellum contributes to perceptual processes.

Dr. Schmahmann sets the stage for functional hypotheses. Inspired by the cerebellum’s uniform neuroanatomical structure and dense heterogeneous connectivity, he argues that we should assume a constant computation—the universal cerebellar transform—that is applied to multiple domains of neurological function determined by cerebellar connections. The idea of a uniform computation is repeated in many of the other commentaries, although the specific form of the computation shows considerable variation. Building on comparative data from across the animal kingdom, Dr. Paulin suggests that the cerebellum provides the ability to predict state trajectories of dynamical systems. The ability to predict state trajectories of the body and external targets is essential for agile motor control and can explain the obvious, classical symptoms of cerebellar dysfunction. But state estimation can also provide core capability for a variety of signal processing, decision-making and control tasks, and this could explain newer evidence about the cerebellum’s role in non-motor tasks. The latest neuroimaging evidence for direct interaction between the cerebellum and temporal areas involved in visual motion processing and body motion processing (MT/MST and STS), as presented by Drs. Baumann, Mattingley, Pavlova and Sokolov, appears to lend further support to this hypothesis. Similarly, Dr. Ivry’s hypothesis proposes a contribution of the cerebellum to the analysis and prediction of sensory event timing in the sub-second range. Drs. Leggio and Molinari’s hypothesis of the cerebellum’s role in perception shares the central assumption that the cerebellum is involved in the analysis and prediction of dynamic perceptual events. While Dr. Ivry focused here on a narrower view of prediction, events requiring precise timing in the sub-second range, Drs. Leggio and Molinari take a broader view of prediction with their hypothesis that the cerebellum supports perception by extracting sequential order information from incoming sensory information. Clinical and neuroimaging studies not only implicate the cerebellum in the analysis of dynamic stimuli, but also in less dynamic perceptual tasks such as pitch discrimination and nociception. Dr. Bower urges us to consider that the cerebellar contribution arises at an even earlier stage of processing, arguing that the cerebellum influences perception by controlling the acquisition of sensory data, an idea that might explain why cerebellar activity often increases with the difficulty of a perceptual task.

While some of the described theories could be seen as complementary, the challenge remains to develop more explicit experimental tests that can distinguish between these hypotheses. Most of the current evidence is delivered by human lesion and neuroimaging studies, methods that have provided valuable insights from a systems-level perspective, but are of limited value in constraining models at the level of microcircuitry. It is therefore essential to also explore the cerebellum’s involvement in perceptual tasks at the level of single neurons. Dr. Cullen’s research on the role of the cerebellum in self-motion perception provides a compelling example. By recording from individual cerebellar neurons, her research has shown that the cerebellum computes sensory prediction error signals that effectively distinguish between the sensory consequences of self-generated and externally produced actions. These findings seem inconsistent with the conventional view that the role of the cerebellum is restricted to motor learning.

Finally, an important application of new knowledge arising from research into the role of the cerebellum in perception is in the domain of human health and disease. The historical association of the cerebellum with “motor function” has limited appropriate consideration of its potential role in perceptual functions, in both health and disease. It is now apparent that cerebellar lesions can lead to a range of behavioral, cognitive, affective, and perceptual impairments. In addition, psychiatric conditions that are characterized by perceptual and cognitive (as well as motor) disturbances, including autism, schizophrenia, and attention deficit hyperactivity disorder, are associated with cerebellar pathology. The possibility of a cerebellar role in the manifestations or pathogenesis of these conditions is intriguing. Further research into the role of the cerebellum in perceptual functions may help to advance our understanding of the mechanisms underlying these disorders. Moreover, patients with isolated cerebellar insults, cerebellar tumors, and hereditary cerebellar degenerative disease will also benefit from a better understanding of the role of the cerebellum in perception. To date, diagnostic evaluation and therapeutic interventions in patients with cerebellar disease have been limited to the striking deficits in the coordination of voluntary movements. Recognition of a cerebellar role in sensory processes helps to identify and treat potential perceptual deficits that may at present go unnoticed and untreated. In addition, further research on the compensatory potential of not only motor, but also perceptual cerebro-cerebellar networks after cerebellar damage may advance both clinical management and understanding of the cerebellar contribution to perception.

This review is the first attempt to capture the variety of current experimental approaches and theoretical models on the cerebellum’s role or influence on perception. By drawing together the diverse perspectives, we intend to stimulate scientific debate and increase interest in the cerebellum and its complex functions.
